# High-Efficiency Plasma Source Using a Magnetic Mirror Trap for Miniature-Ion Pumps

**DOI:** 10.3390/s23021040

**Published:** 2023-01-16

**Authors:** Yuichi Kurashima, Taisei Motomura, Shinya Yanagimachi, Takashi Matsumae, Mitsuhiro Watanabe, Hideki Takagi

**Affiliations:** 1 Device Technology Research Institute, National Institute of Advanced Industrial Science and Technology, Ibaraki 305-8564, Japan; 2 Sensing System Research Center, National Institute of Advanced Industrial Science and Technology, Saga 841-0052, Japan; 3 Research Institute for Physical Measurement, National Institute of Advanced Industrial Science and Technology, Ibaraki 305-8563, Japan; 4 College of Science and Technology, Nihon University, Chiba 274-8501, Japan

**Keywords:** miniature ion pump, ultra-high vacuum, anodic bonding, laser cooling atom

## Abstract

In this study, we design a highly efficient plasma source using a magnetic mirror trap with two opposing permanent magnets for a miniature high-efficiency ion pump. First, we simulated the distribution of the magnetic field line formed by the proposed magnetic mirror configuration. By optimizing the distance between two opposing permanent magnets and size of these magnets, a magnetic mirror ratio value of 27 could be obtained, which is an electron confinement efficiency of over 90%. We also conducted an experiment on a high-efficiency discharge plasma source for a miniature ion pump using an optimized magnetic circuit. As a result, we revealed that the proposed magnetic circuit has a pronounced effect on plasma generation, particularly in the high-vacuum region.

## 1. Introduction

Various microdevices, such as microelectromechanical systems (MEMS), require hermetic sealing to protect their mechanical structure from an external environment and to ensure device performance [1,2,3,4]. After degassing by vacuum annealing, hermetic sealing is performed in a vacuum or inert gas. A getter is also placed in the cavity for higher-level hermetic sealing and activated to adsorb the residual gas [5,6]. In recent years, the miniaturization of devices for physical phenomena in ultra-high vacuum, such as laser cooling atoms and the ion trapping phenomenon, has been required [7,8,9,10,11]. However, ultra-high vacuum cannot be realized using the conventional hermetic sealing technologies described above. Whereas nonevaporable getters have been developed for ultra-high vacuum (UHV) conditions, it is difficult to adapt them to chip-scale cells because of the inability to evaluate the inside pressure with accuracy [12]. Therefore, it is important to develop a miniature ultra-high vacuum pump that can actively evacuate the inside cavity space of a chip-scale cell after hermetic sealing. Ion pumps, turbomolecular pumps, and cryogenic pumps are candidates for ultra-high vacuum pumps. Among them, an ion pump can be realized on a chip scale by the MEMS process, which is appropriate for the devices. The ion pump works not only as a vacuum pump but also as a Penning vacuum gauge; it consists of two magnets, an anode, and two titanium cathodes positioned outside the anode [13]. A cold cathode discharge occurs in a magnetic field when a high voltage is applied. Electrons emitted from the electrodes make a round trip between the cathodes with helical motion owing to the Lorenz force in the magnetic field. When the electrons collide with gas molecules, the gas molecules become ionized, that is, they become ions. These ions collide with the cathode surface and sputter the titanium atoms. The sputtered Ti atoms form a clean Ti film, called a getter film, on the anode, cathode, and inner walls of the pump. Because sputtered titanium atoms are chemically active, they adsorb residual gas molecules. Some of the incident ions are also embedded in the cathode. Therefore, the ion pump is also effective for inactive gases. As a result, the pump can realize ultra-high vacuum conditions in a chamber. Scott et al. developed a chip-scale Penning cell array for sputter ion pumping by assembling several machined parts [14]. Recently, Grzebyk et al. developed a miniature ion pump fabricated by the anodic bonding of Si and glass, which results in a hermetically sealed cell. They realized a high-vacuum chip-scale cell using their developed miniature ion pump [15,16,17,18].

However, because of the narrow electrode gap of a few millimeters in the case of the miniature ultra-high vacuum pump, the potential gradient becomes too high. Therefore, electrons move from the cathode to the anode in a straight way with a short trajectory, and the collision rate of electrons with gas particles drastically decreases in the high vacuum range. Increasing the flight distance of electrons is important for achieving a high discharge efficiency. Grzebyk et al. also developed a miniature ion source based on the magnetron structure, which operates in a wide pressure range from low to high vacuum [19]. The magnetron-like miniature ion source was realized by the MEMS micro fabrication process. In this study, we propose a high-efficiency plasma source with a magnetic mirror configuration using opposed permanent magnets. Our proposed plasma source can realize a high-efficiency plasma by only adjusting the arrangement of magnets.

## 2. Design, Simulation, and Experimental

**Figure 1:**
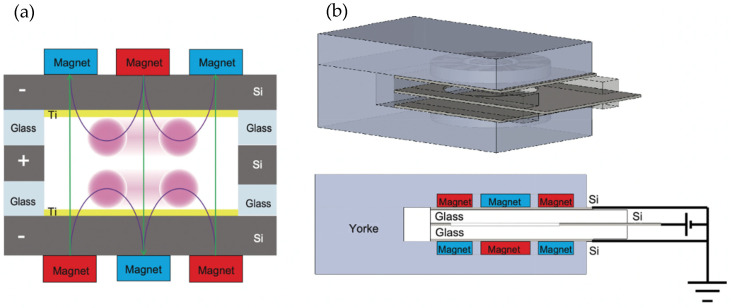
(**a**) Schematic cross-section image of the magnetic circuit for highly efficient plasma production. (**b**) Designed magnetic circuit and electrodes.

Figure 1a shows the layout for two pairs of opposed permanent magnets for the magnetic mirror configuration and predicted magnetic field lines. Ring-shaped permanent magnets were placed outside cylindrical permanent magnets. These two pairs of permanent magnets were placed opposite each other, as shown in Figure 1a. The cylindrical permanent magnets have a thickness and diameter of 2 and 7.2 mm, respectively. The ring-shaped permanent magnets have an outside diameter, internal diameter, and thickness of 20, 9.6, and 2 mm, respectively. Silicon electrodes were installed inside the magnetic circuit as plasma sources. A silicon electrode with dimensions of 34 × 24 × 0.4 mm^3^ and a hole of 16 mm in diameter was used as the anode. Two silicon electrodes with dimensions of 24 × 24 × 0.4 mm^3^ were placed on both sides of the anode as cathodes. The inner surfaces of the cathode silicon electrodes were covered with thin Ti layers, and the silicon electrodes were electrically insulated using borosilicate glass with dimensions of 4 × 29 × 2 mm^3^. The silicon and borosilicate glass were bonded by anodic bonding. 

The magnetic field line distributions inside the magnetic circuit were calculated at different magnet pair distances ranging from 1 to 20 mm using the 2D finite element method. The magnetic field line distributions were also simulated at a magnet pair distance of 5 mm for different magnet thicknesses. For the simulations and discharge experiments, we used readily available neodymium magnets with a residual flux density of 1230 mT and a coercive force of 836 kA/m, both of which are high among permanent magnets. The electron trajectories inside the electrodes were calculated by 3D finite element analysis using a magnetic circuit with a magnetic mirror configuration. For the simulation, 500 V was applied to the anodic electrode in vacuum.

For the discharge experiments, the distance between two sets of opposed permanent magnets was set as 5 mm, which was based on numerical simulations described in this report. The magnets were additionally covered with a yoke (SS400), as shown in Figure 1b. The other details of the magnetic circuit and electrodes were as described at the beginning of this chapter. The fabricated plasma source was then placed in a vacuum chamber. A discharge voltage was applied using a vacuum feedthrough. The relationship between the discharge current and the cathode-anode voltage was measured for different pressures. The dependence of the ignition voltage on pressure was evaluated for two different types of magnetic circuits.

## 3. Result and Discussion

**Figure 2:**
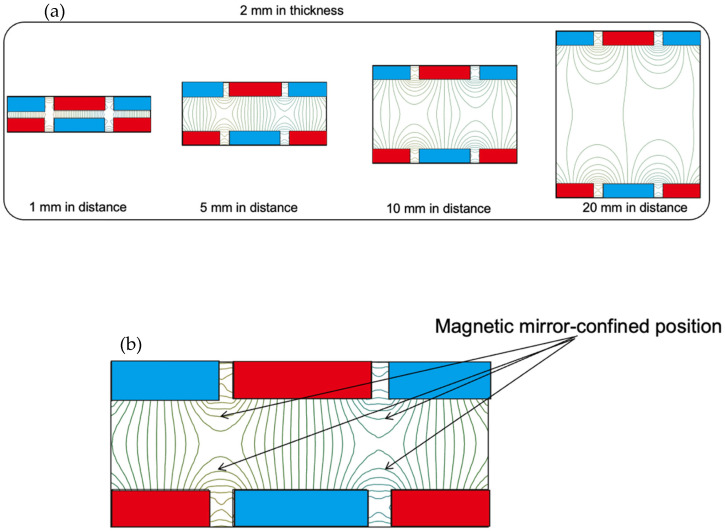
(**a**) Magnetic field lines inside the magnetic circuit calculated by the 2D finite element method for magnets with different distances. (**b**) Magnetic field lines inside the magnetic circuit in the case of 5 mm in distance.

Figure 2 shows the magnetic field lines inside the magnetic circuit, calculated using the 2D finite element method. With 2-mm thick magnets and 1 mm of distance between the magnets, the parallel magnetic field component (longitudinal direction on the paper) is dominant between the magnets, and we cannot see the magnetron magnetic field component (transverse direction on the paper) in the plasma production region. In contrast, the magnetron field component is dominant over the parallel field component at a distance of 20 mm between the magnets. Both parallel and magnetron magnetic field components were present in the cases with 5 and 10 mm between the magnets. Charged particles are trapped at locations of low magnetic field strength along the magnetic field lines. Therefore, it seems that the charged particles can be confined at four locations at a distance of 5 mm between the magnets with magnetron and parallel magnetic field components, as shown in Figure 2b. For the case of 5 mm in distance between the two sets of magnets, the magnetic mirror ratio was calculated to be 27, resulting in an electron confinement efficiency greater than 90%. 

**Figure 3:**
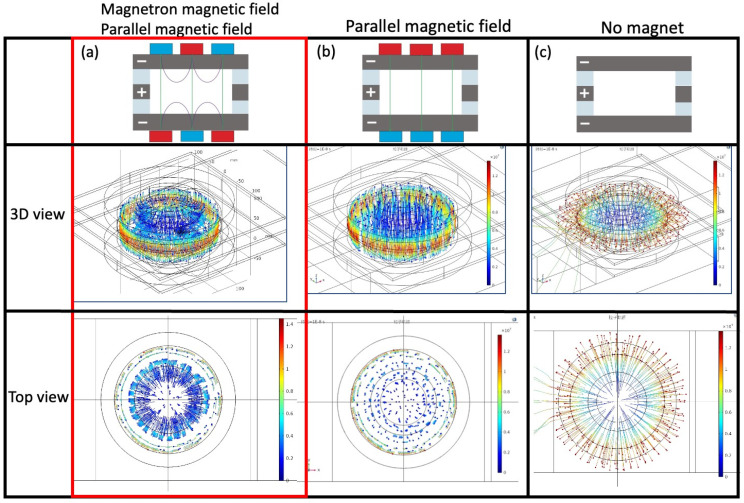
Electron trajectory calculated by 3D finite element analysis for three different magnetic circuits.

Figure 3 displays electron trajectories calculated by 3D finite element analysis for three different magnetic circuits. In the case of the magnetic circuit with magnetic mirror configuration as shown in Figure 3a, electrons are reciprocated in the poloidal (small circumferential) direction by the plasma confinement effect of the magnetron magnetic field. The electrons are orbitally rotated in the toroidal (large circumferential) direction by the $E_{\|}\times B_{⊥}$ drift motion, where $E_{\|}$ is the parallel electric field component induced to Si electrodes, and $B_{⊥}$ is the transverse magnetron magnetic field component. In a magnetic circuit with only a parallel magnetic field, the electrons move with an up and down cyclotron motion between these cathode electrodes along the parallel magnetic field and electrostatic force, as shown in Figure 3b. This electron behavior is the same as that of a conventional ion pump. Figure 3c shows the electron behavior inside electrodes without a magnet. In this case, the electrons only move up and down rectilinearly between these cathode electrodes. Based on the above simulated results, plasma confinement can be realized by the proposed magnetic circuit with the magnetron and parallel magnetic fields.

**Figure 4:**
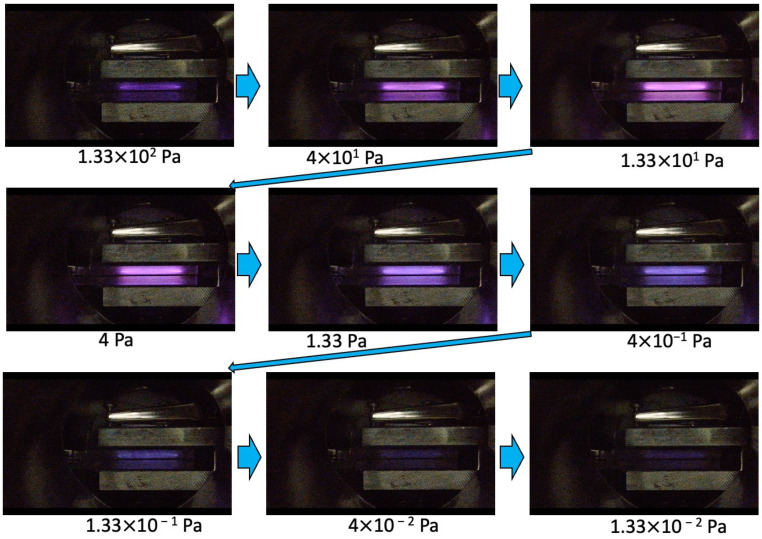
Pictures of glow discharge inside the electrodes at each pressure.

Figure 4 exhibits pictures of the glow-discharge from using the proposed miniature ion source in a low vacuum range from 133 to 133 × 10^−2^ Pa. These pictures were taken near the ignition voltage at each pressure. Bright parts by discharge can be seen at the positions of electron confinement, which are consistent with the high-density electron confinement positions calculated in the above simulation (Figure 3a). These discharge colors were changed from violet to pink and violet and then to dark violet. The brightness decreases with decreasing vacuum chamber pressure in the low Pa region along with the decrease of discharge current. Whereas the discharge light was barely visible below 1.33 × 10^−2^ Pa, tiny discharge currents were measured below 100 μA.

**Figure 5:**
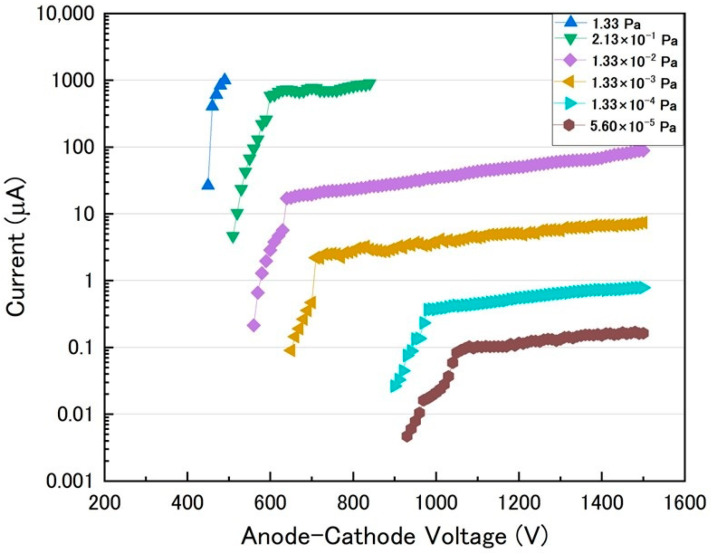
Discharge current depending on anode-cathode voltage for different pressures.

Figure 5 shows the discharge current depending on the anode-cathode voltage for different pressures. The applied voltage was increased by 10 V each from voltage sufficiently lower than the ignition voltage. At a pressure of 1.33 Pa, the current was suddenly increased from the applied voltage of 450 V. We defined an ignition voltage as a value at which the current increased suddenly. Voltage was applied up to 490 V between the anode and cathode due to the set current limitation of 1 mA. The ignition voltages increased with decreasing pressure. At a pressure of 5.6 × 10^−5^ Pa, the ignition voltage was 930 V. After ignition, the discharge current increased sharply with the application of increasing voltage up to approximately 1050 V. In addition, the discharge current exponentially increased with the application of increasing voltage over 1050 V. Although ignition began at an applied voltage of 930 V, the discharge may have been unstable because the plasma was not sufficiently confined by the mirror magnetic field in the range of applied voltage, approximately 930 to 1050 V. In contrast, when the applied voltage was higher than 1050 V, the current increased gradually with increasing voltage. This result suggests that the plasma is effectively confined to a small cavity by the proposed mirror magnetic field. 

**Figure 6:**
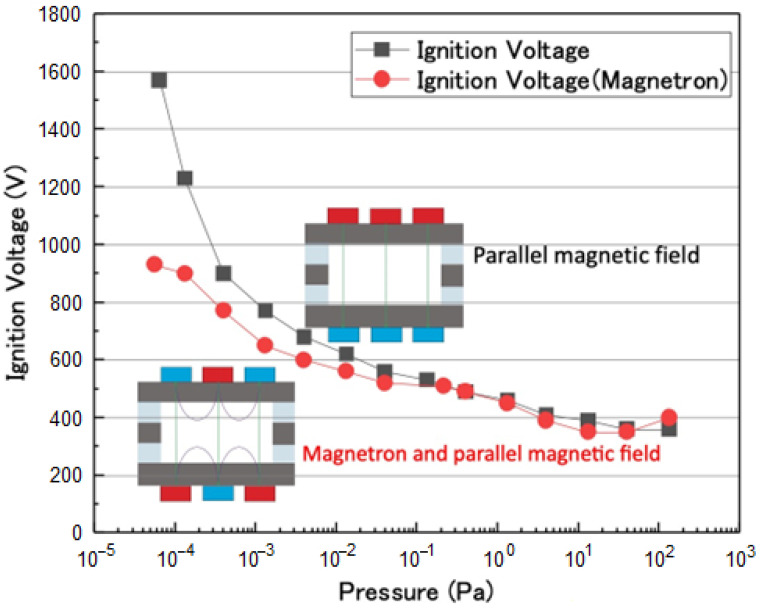
Ignition voltage depending on pressure for two types of magnetic circuits.

Figure 6 shows the ignition voltage depending on pressure for two types of magnetic circuits. Red circles and black squares represent the ignition voltages for the plasma source using the proposed mirror magnetic and parallel magnetic fields, respectively. In the case of a pressure higher than 10^2^ Pa, discharges occurred outside of the magnetic circuit. The two graphs exhibit similar behavior in the range of 10^−1^ to 10^2^ Pa. In contrast, the ignition voltage was lower for the plasma source using the proposed mirror magnetic field compared to that of the parallel magnetic field at pressures lower than 10^−2^ Pa. This trend of ignition voltage appeared prominently in a region of ultra-high vacuum. An ignition voltage of 1570 V was required for the plasma source using the parallel magnetic field at 6.4 × 10^−5^ Pa. In contrast, the ignition voltage was 930 V for the proposed mirror magnetic field at a pressure of 5.6 × 10^−5^ Pa, approximately 56% lower than that of the parallel magnetic field. This result suggests that the mirror-confined plasma in the proposed mirror magnetic field has a pronounced effect on plasma generation, and a high-efficiency plasma source was realized, particularly for an ultra-high vacuum region.

## 4. Conclusions

We proposed a high-efficiency plasma source using a magnetic mirror configuration for a miniature ion pump. From the simulation of electron trajectories calculated by 3D finite element analysis, electrons were reciprocated in the poloidal (small circumferential) direction by the plasma confinement effect of the magnetron magnetic field. The electrons were rotated in the toroidal (large circumferential) direction by the $E_{\|}\times B_{⊥}$ drift motion. Plasma confinement was realized using the proposed magnets with magnetron and parallel magnetic fields. The discharge characteristics of the proposed plasma source were also evaluated. The ignition voltage was lower for the plasma source using the proposed mirror magnetic field compared to that of the conventional parallel magnetic field at pressures lower than 10^−2^ Pa. Therefore, we conclude that a high-efficiency plasma source can be realized using the proposed magnets with magnetron and parallel magnetic fields.

## Data Availability

The data presented in this study are available on request from the corresponding author.
